# Factors affecting the development of cystoid macular edema following pars plana vitrectomy with silicone oil placement: a retrospective cohort study

**DOI:** 10.1186/s40942-024-00582-0

**Published:** 2024-09-12

**Authors:** Reem H. ElSheikh, Sairi Zhang, Muhammad Z. Chauhan, Riley N. Sanders, Sami H. Uwaydat

**Affiliations:** 1 Department of Ophthalmology, Harvey and Bernice Jones Eye Institute, University of Arkansas for Medical Sciences, 4301 W. Markham Street, Slot # 523, Little Rock, AR 72205 USA; 2 College of Medicine, University of Arkansas for Medical Sciences, Little Rock, AR USA

**Keywords:** Cystoid macular edema, Retinal detachment, Silicone oil, Pars plana vitrectomy, Scleral buckle, Surgical outcomes

## Abstract

**Background:**

Cystoid macular edema (CME) can develop following silicone oil placement in complex vitreoretinal surgeries, contributing to poor visual outcomes. In this study, we investigated the clinical and surgical characteristics associated with the development of CME following the use of silicone oil (SO) in pars plana vitrectomy (PPV) surgeries.

**Methods:**

We conducted a retrospective chart review of patients who underwent implantation of SO during PPV from 2010 to 2020 by a single surgeon. Patient demographics, type of oil, duration of oil tamponade, retinectomy size, diabetic status, lens status, prior panretinal photocoagulation, visual acuity, and incidence of CME were reviewed.

**Results:**

This study included 43 eyes from 40 patients who underwent SO tamponade for retinal detachment (RD) surgery. The mean duration of SO tamponade was 15.7 ± 12.7 months (range: 1–58 months). The most common indication for surgery was diabetic tractional RD (32.7%), followed by traumatic RD (16.3%) and rhegmatogenous RD with proliferative vitreoretinopathy (11.6%). Of the 43 eyes, 18 (41.9%) developed CME for the first time after PPV with SO placement, with 8 (44%) resolving within a year of oil removal. The mean duration for the development of CME was 9 months. A logistic regression model showed that a scleral buckle procedure and poor initial vision were statistically significant factors for predicting the development of CME (ORs: 11.65 and 16.06, respectively). Overall, 91% of the patients had stable or improved vision after surgery.

**Conclusions:**

The use of a scleral buckle procedure and poor initial vision are significant factors for predicting CME following silicone oil tamponade in PPV surgeries, with 41.9% of patients developing CME with an average duration of 9 months. Recognizing such factors can lead to early monitoring and prompt management of CME.

**Meeting presentation:**

Partial analyses were presented at the ASRS 2020 conference.

**Clinical trial number:**

Not applicable.

## Background

In pars plana vitrectomy (PPV), intraocular tamponade is often required to reduce postoperative retinal detachment recurrence [1]. The choice of whether to use gas or silicone oil (SO) usually depends on several factors including the size and configuration of the retinal detachment (RD), presence and position of retinal tears, lens status (phakic, aphakic, or pseudophakic), and patient requirement for air travel in the postoperative period [2]. SO was first used in 1962, and the scale of its use has since been extended to include RD associated with giant tears, trauma, uveitis, macular holes, and recurrent detachments [1, 3, 4]. It has also been used in proliferative diabetic retinopathies and proliferative vitreoretinopathy (PVR).

Although SO has yielded great anatomical and functional outcomes, its efficacy has been hindered by substantial side effects including secondary cataracts, increased intraocular pressure, chronic hypotony, and emulsification into the anterior chamber with subsequent band keratopathy and corneal decompensation [3, 5, 6]. Some cases showed inadequate visual outcomes despite postoperative anatomical success. This may be due to the presence of cystoid macular edema (CME), retinal folds or epiretinal membranes, or, in some cases, persistent foveal detachment despite retinal reattachment. CME can have a significant impact on the patient’s visual outcome, even when the surgical procedure is otherwise successful. Therefore, it is crucial to understand the factors that contribute to the development of CME and prevent it from occurring.

In a study conducted by Yang and colleagues [7], different factors such as axial length, duration of SO in the eye, lens status, and posterior staphyloma were evaluated to determine the risk factors for CME after silicone oil tamponade for retinal detachment. The presence of posterior staphyloma was associated with the development of CME. The current study aimed to examine a broader range of patient-level and surgery-related factors that may influence the development of CME following the use of silicone oil in PPV.

## Methods

In our study, the charts of patients who underwent SO placement during PPV by a single surgeon at the University of Arkansas for Medical Sciences between January 1, 2010, and January 31, 2020, were retrospectively reviewed. The data collected included patient demographic characteristics including age, gender, and diabetes status. Preoperative visual acuity (VA) and the presence of preexisting macular edema or other retinal pathologies were recorded. Surgical details including internal limiting membrane (ILM) peeling, scleral buckle (SB) placement, preexisting panretinal photocoagulation (PRP), concurrent cataract extraction (CE), retinectomy, indication for SO injection, type of SO used (1000 or 5000 centistokes), and retinectomy size were collected from the operative reports. The retinectomy size was categorized based on quadrants of the excised tissue.

Postoperative data collected included the time of CME development as indicated by optical coherence tomography (OCT), management of CME, and patient outcomes. CME was evaluated while patients had silicone oil and after oil removal when the information was available. The primary outcome of the study was the proportion of patients who developed CME for the first time after PPV with silicone oil placement, and the main objective was to investigate factors associated with CME development. Secondary outcomes included VA change, surgery-related complications, and the need for further medical or surgical treatment related to the retinal condition. An initial TriNetX search at our institution of procedure codes for complex retinal detachment surgery with SO tamponade revealed up to 290 potential patient charts to review. The inclusion criteria included patients who had undergone PPV with SO tamponade for advanced retinal pathologies (rhegmatogenous RD, diabetic tractional RD, trauma, retinal breaks, giant retinal tears) and had at least 6 months of follow-up data. We excluded patients who had preexisting conditions contributing to the presence of CME, such as diabetic macular edema and retinal vein occlusion. Sixty-three eyes of 59 patients were included in our study.

Descriptive data were expressed as the mean, standard deviation, and range values. The primary analysis included a logistic regression model to assess factors associated with the development of CME. The independent variables included age, gender, lens status (aphakic, phakic, and pseudophakic), type of SO used, retinectomy size (quantified as a categorical value (1,2,3,4) based on the quadrants of the excised tissue), initial logMAR VA, best logMAR VA under oil, number of intraoperative complications, postoperative ocular hypertension, SB procedure performed, ILM removal, and preexisting PRP. Logistic regression analysis was used to determine patient- and surgical-related factors for the development of CME after SO use. All analyses were performed using Stata Release 17.0 (StataCorp LLC). Tests with 2-sided P values less than 0.05 were considered to indicate statistical significance.

## Results

We reviewed 63 eyes of 59 patients (4 patients had bilateral SO injections). Twenty eyes were excluded due to inadequate follow-up criteria, leaving 43 eyes from 40 patients in the final analytical dataset. The mean age of the patients included in the study was 56.5 ± 14.7 years (range: 13 to 85 years) with 19 females (47.5%) and 21 males (52.5%) (Table 1). The mean follow-up duration was 36.1 ± 22.0 months. The indications for surgery varied with 32.7% (14 eyes) having diabetic tractional RD, 16.3% (7 eyes) having traumatic RD, and 11.6% (5 eyes) having rhegmatogenous RD with PVR. Seventeen eyes (39.5%) underwent surgery for less common indications including schisis RD, RD from a macular hole, rhegmatogenous RD from a giant retinal tear, and posttraumatic and endogenous endophthalmitis. Regarding lens status, 67.4% (29 eyes) of the patients were pseudophakic, 25.6% (11 eyes) were aphakic, and 7.0% (3 eyes) were phakic. The mean duration of SO tamponade was 15.7 ± 12.7 months (range: 1–58 months). Intraoperative complications included suprachoroidal hemorrhage in 1 patient, iatrogenic retinal breaks in 2 patients, and a macular hole in 1 patient.

**Table 1:** Characteristics between patients with and without cystoid macular edema following PPV

Characteristics	Non-CME *(* *n* * = 23 Pts.; 25 Eyes)*		CME *(* *n* * = 17 Pts.; 18 Eyes)*		*P*-value^*^
	*By Patient*				
**Sex (No.**,** %)**					0.491
*Female*	12 (52.17)		7 (41.18)		
*Male*	11 (47.83)		10 (58.82)		
**Age (Mean**,** SD)**	54 (16.25)		59.88 (11.95)		0.215
**Follow-Up in Months (Mean**,** SD)**	40.55 (25.32)		30.82 (16.53)		0.184
	*By Eye*				
**Lens Status (No.**,** %)**					0.344
*Aphakic*	5 (20.00)		6 (33.33)		
*Phakic*	1 (4.00)		2 (11.11)		
*Pseudophakic*	19 (76.00)		10 (55.56)		
**Initial logMAR VA (Mean**,** SD)**	1.92 (0.67)		2.28 (0.340)		**0.044**
**Best logMAR VA Under Oil (Mean**,** SD)**	1.78 (0.68)		1.74 (0.62)		0.832
**Pre-existing PRP (No.**,** %)**	12 (48.00)		8 (44.44)		0.818
**History of Diabetes (No.**,** %)**	14 (56.00)		6 (33.33)		0.142
**Scleral Buckle (No.**,** %)**	12 (48.00)		13 (72.22)		0.112
**Intraoperative Complications (No.**,** %)**	3 (12.00)		1 (5.56)		0.423
**ILM Removed (No.**,** %)**	14 (56.00)		12 (66.67)		0.480

Note. CME, cystoid macular edema; PPV, pars plana vitrectomy; ILM, internal limiting membrane; SD, standard deviation, No., frequency. *T-test of continuous and Chi-square for categorical variables

Our patient review showed that 41.9% (18 eyes) of patients developed CME for the first time under oil. The mean duration for the development of CME was 9 months. Of the 18 eyes that developed CME for the first time under oil, CME resolved within a year of removal of oil in 8 eyes (44%), while 10 eyes (56%) had persistent CME for more than 1 year. Initial treatment consisted of a combination of topical prednisolone acetate and ketorolac. Subtenon kenalog or intravitreal triamcinolone injection was required in 2 patients with poor or no response to topical treatment. The average duration to the resolution of CME was 19 ± 17.21 months. Of the 43 eyes in our study, 3 were re-detached after the SO was removed and 9 were re-detached under the SO. Regarding visual outcomes, 91% (39 out of 43 eyes) had a stable or better VA than that recorded preoperatively.

**Fig. 1:**
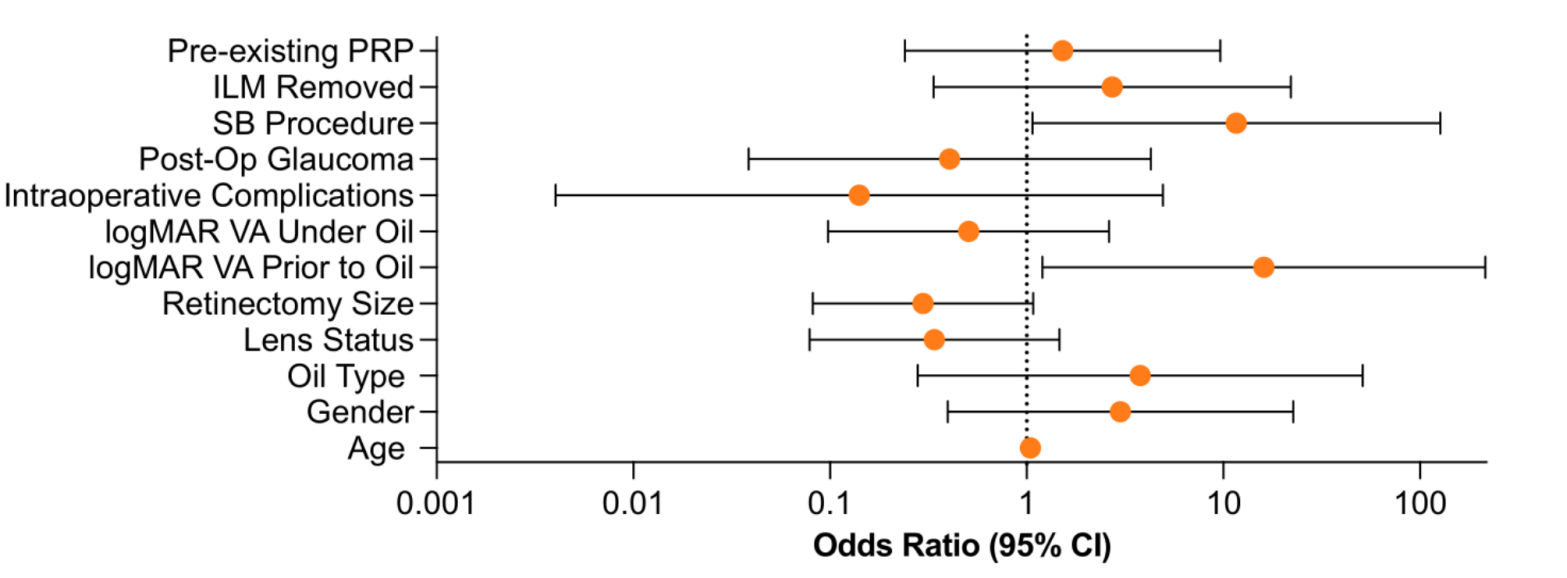
Odds ratios and 95% confidence intervals from the logistic regression model for the development of first-time CME post-PPV with SO placement

We subsequently constructed a logistic regression model to determine the factors associated with the development of CME for the first time under oil. Multivariate analysis revealed that a scleral buckle procedure significantly predicted the development of CME under oil (odds ratio (OR), 11.65, *p* = 0.044). We also found that worse initial vision was associated with the development of CME (OR, 16.06, *p* = 0.036) (Fig. 1).

## Discussion

During retinal surgeries, procedures such as subretinal fluid drainage, diathermy, endolaser, cryotherapy, and scleral buckle are injurious by nature and lead to the release of inflammatory mediators and subsequent development of CME. This is supported by the increased levels of inflammatory cytokines found in aqueous samples from patients who had undergone PPV [8, 9]. A study conducted by Chatziralli and colleagues revealed that the presence of preoperative PVR, a long duration of rhegmatogenous RD, and macula-off RD were all factors that correlated with the presence of CME after PPV [10]. All the previously mentioned factors are associated with inflammation and the release of inflammatory cytokines [11, 12]. PVR membranes are formed in response to the release of inflammatory cytokines after tissue damage and inflammation caused by retinal detachment. Another study by Merad and colleagues revealed that preoperative severe grade C PVR and low preoperative best-corrected visual acuity (BCVA) were significantly associated with CME after PPV [13]. Additionally, Starr and colleagues reported that successful single retinal surgery was associated with a lower incidence of postoperative CME (less inflammation), while preoperative PVR was associated with a higher incidence of CME in PPV patients [14]. 

During SO placement, a persistent retro-oil space is created since the eye is not completely filled with oil. A study conducted by Asaria and colleagues [15] sampled intraocular fluid and vitreous samples from eyes that had undergone vitreoretinal surgeries and found a greater concentration of inflammatory mediators, including protein, interleukin-6, transforming growth factor beta, and fibroblast growth factor, in the retro-oil space when compared to all other samples. This may promote the development of CME in these patients. Another study showed the resolution of CME after SO removal, hypothesizing that the CME was due to the free dispersion and diffusion of these trapped mediators into the vitreous cavity [16]. 

The aim of this study was to investigate the factors related to the development of CME and whether the presence and size of retinectomy during PPV correlate with the subsequent development of CME. After performing multivariate logistic regression to account for confounding variables, CME development was found to be significantly associated with worse initial VA and the use or presence of a scleral buckle.

Other factors, such as ILM removal, SO type, diabetes status, preexisting PRP, and retinectomy size, did not correlate with the development of CME. ILM peeling has been shown to reduce macular edema by improving the oxygenation of the inner retina [17]. The presence of silicone oil in the vitreous may have prevented the inner retinal oxygenations in patients who had their ILM peeled. Both diabetes and PRP can alter the blood retinal barrier and are independent risk factors for developing CME after vitrectomy [18], though our study did not find a correlation of either with CME. Stopa et al. found that 33% of patients who underwent retinectomy for repair of RD with PVR developed CME, but the retinectomy size did not influence the incidence of CME [19]. The need for intraoperative retinectomy is an indication of a severe retinal pathology with increased intraocular inflammation. In our cohort, we did not find an association with CME and retinectomy size, which we attribute to the effect of silicone oil on the intraocular compartmentalization.

The incidence of CME after PPV with the use of SO tamponade in our study was 42%. This is higher than that reported in previous studies, with incidences ranging between 14% and 36% [7, 20] Yang and colleagues reported an incidence of 36% for CME after vitrectomy with SO tamponade. They related the relatively high incidence to the longer duration that SO was kept in the eye compared to previous studies (276 and 273 days in the CME and non-CME groups, respectively (*p* = 0.96)). In Yang’s study, CME occurred after an average of 171 days postoperatively, while in our study, CME occurred after an average of 9 months (274 days) after oil placement. Other studies concluded that the duration of SO did not correlate with the development of CME [7]. Although there is currently no fixed optimal timing for SO removal, the consensus is that removal should occur within 3 to 6 months to avoid associated complications.

In our study, there was a statistically significant correlation between worse initial VA (higher logMAR) and the development of CME. Similarly, in the studies conducted by Merad et al. and Berrod et al., worse initial BCVA was found to be associated with the development of CME. A worse initial BCVA usually indicates a worse initial pathology of RD. Such pathologies are associated with increased inflammation and the resultant release of inflammatory mediators that contribute to the pathology of CME [13]. 

Our study also showed that the presence of a scleral buckle was significantly associated with the subsequent development of CME. The use of a scleral buckle also indicates a more severe initial pathology in addition to creating a more complex surgery. Both poor initial VA and presence of a scleral buckle are associated with an increase in the inflammatory process and the subsequent development of CME. Pole and colleagues also found that the number of surgeries contributed to the development of CME such that eyes with CME had a higher number of surgeries, although the difference did not reach statistical significance [21]. In contrast, Shah et al. determined that a high number of total retinal detachment surgeries was significantly associated with CME development, in addition to prior intraocular surgery and the development of an epiretinal membrane (ERM) after surgery [22]. Gebler et al. also found that the presence of ERM significantly increased the risk of postoperative CME [23]. 

A study conducted by Frisina and colleagues [24] evaluated different factors correlating with the development of CME after PPV. The study concluded that the prevalence of CME was 6.03% versus 5.17% in the phakic and pseudophakic patient groups, respectively. The difference was not statistically significant. Banker and colleagues [25] also concluded that lens status was not a risk factor for the development of CME after PPV (15.7% in phakic patients versus 14.7% in pseudophakic patients (*p* = 0.75)). In our study, 67% of the patients were pseudophakic, 26% were aphakic, and 7% were phakic. The incidence of CME is generally greater when two procedures are combined in a single surgery, given the complexity of the surgery. Pole and colleagues concluded that complex retinal detachment surgeries (*p* < 0.001) as well as pseudophakic and aphakic lens status (*p* = 0.008) were significantly associated with the development of postoperative CME [21]. The higher incidence of CME in our study may be due to the actual lens status (aphakic) resulting in a unicameral circulation of inflammatory cytokines within the eye, the complexity of the surgery that included both PPV and cataract extraction, or the complexity of the retinal pathology that required keeping the patient aphakic. Chatziralli and colleagues found that pseudophakic lens status was associated with postoperative CME in PPV patients [10]. Since this point is currently debatable, it could be beneficial to study the effect of performing cataract extraction before PPV and compare results between the groups of patients.

Pole and colleagues reported that the rate of retinectomy was higher in the group that developed CME than in the non-CME group (36% (9 out of 25) versus 5% (4 out of 74)) (*p* = 0.0009), which was attributed to the complexity of the surgical procedure. Our study found no statistically significant correlation between retinectomy or retinectomy size and the development of CME.

Other factors not included in our current study that have been linked to the development of postoperative CME in PPV patients include posterior staphyloma but not axial length [7]. Other studies have shown that axial length (*p* < 0.005), the use of cryopexy, a longer duration of macular detachment, and a history of posterior capsular rupture are all related to the development of CME in postoperative PPV patients [26]. 

In the study by Yang and colleagues, 13 out of 21 eyes with CME had undergone SO removal. Among these 13 eyes, 11 had complete resolution of CME, while two did not. Nine eyes recovered spontaneously without needing further intervention, one eye received an intravitreal anti-vascular endothelial growth factor (VEGF) bevacizumab injection (Avastin^®^, Genentech/Roche, South San Francisco, California), and one eye received a posterior sub-tenon triamcinolone injection (PSTI) with an overall recovery time of 70 days on average. In other studies evaluating CME after PPV using SO tamponade, Bae and colleagues [16] demonstrated that 8 out of 9 eyes achieved complete resolution of CME within 6 months of SO removal irrespective of ILM peeling during SO removal. Kaharan and colleagues [20] noted a complete resolution of CME in 3 eyes within one month of SO removal. Thus, most prior studies have demonstrated complete resolution of CME within the first year after SO removal without additional intervention. In our study, 47.2% of eyes (17 eyes) that developed CME were managed expectantly with observation only, while other patients were treated with a combination of nonsteroidal anti-inflammatory drugs (NSAIDs), sub-tenon Kenalog injections (STKs), topical steroids, intravitreal triamcinolone, and intravitreal anti-VEGF agents.

Currently, the literature on factors related to postoperative CME in PPV patients is both lacking and, in some cases, conflicting. Although our study revealed a correlation between the development of CME and factors such as a worse initial VA and scleral buckle, it was limited by its retrospective study design and patient selection which may have introduced biases. Other limitations include the relatively small sample size, variability in the etiology of retinal detachment in our study population, and exclusive use of OCT to diagnose CME. Use of other imaging methods such as fluorescein angiography would allow for exclusion of other causes of CME from the differential diagnosis. A prospective study with sufficient follow-up and control groups is needed to establish causality.

## Conclusions

A scleral buckle procedure and poor initial vision are significant factors for predicting CME following silicone oil tamponade in PPV surgeries, with 41.9% of patients developing CME with an average duration of 9 months. However, further prospective randomized studies are needed to highlight the factors that correlate to the development of CME so that these patients can be monitored and managed promptly.

## Data Availability

The datasets used and/or analyzed during the current study are available from the corresponding author on reasonable request.

## Abbreviations

BCVA: Best-corrected visual acuity
CE: Cataract extraction
CME: Cystoid macular edema
ERM: Epiretinal membrane
ILM: Internal limiting membrane
NSAID: Nonsteroidal anti-inflammatory drug
OCT: Optical coherence tomography
PPV: Pars plana vitrectomy
PRP: Panretinal photocoagulation
PSTI: Posterior sub-tenon triamcinolone injection
PVR: Proliferative vitreoretinopathy
RD: Retinal detachment
SB: Scleral buckle
SO: Silicone oil
STK: Sub-tenon Kenalog injection
VA: Visual acuity
VEGF: Vascular endothelial growth factor
